# Bibliometric visualization analysis of gut-kidney axis from 2003 to 2022

**DOI:** 10.3389/fphys.2023.1176894

**Published:** 2023-06-09

**Authors:** Sinan Ai, Yake Li, JiaYin Tao, Huijuan Zheng, Lei Tian, Yaoxian Wang, Zhen Wang, Wei Jing Liu

**Affiliations:** ^1^ Renal Research Institution of Beijing University of Chinese Medicine, Beijing, China; ^2^ Dongzhimen Hospital, Beijing University of Chinese Medicine, Beijing, China; ^3^ Beijing Hospital of Traditional Chinese Medicine, Capital Medical University, Beijing, China; ^4^ Key Laboratory of Chinese Internal Medicine of Ministry of Education and Beijing, Dongzhimen Hospital Affiliated to Beijing University of Chinese Medicine, Beijing, China

**Keywords:** gut microbiota, kidney disease, gut-kidney axis, bibliometric analysis, VOSviewer

## Abstract

**Background:** The gut-kidney axis refers to the interaction between the gastrointestinal tract and the kidneys, and its disorders have become increasingly important in the development of kidney diseases. The aim of this study is to identify current research hotspots in the field of the gut-kidney axis from 2003 to 2022 and provide guidance for future research in this field.

**Methods:** We collected relevant literature on the gut-kidney axis from the Web of Science Core Collection (WoSCC) database and conducted bibliometric and visualization analyses using biblioshiny in R-Studio and VOSviewer (version 1.6.16).

**Results:** A total of 3,900 documents were retrieved from the WoSCC database. The publications have shown rapid expansion since 2011, with the greatest research hotspot emerging due to the concept of the “intestinal-renal syndrome,” first proposed by Meijers. The most relevant journals were in the field of diet and metabolism, such as Nutrients. The United States and China were the most influential countries, and the most active institute was the University of California San Diego. Author analysis revealed that Denise Mafra, Nosratola D. Vaziri, Fouque, and Denis made great contributions in different aspects of the field. Clustering analysis of the keywords found that important research priorities were “immunity,” “inflammation,” “metabolism,” and “urinary toxin,” reflecting the basis of research in the field. Current research frontiers in the field include “hyperuricemia,” “gut microbiota,” “diabetes,” “trimethylamine n-oxide,” “iga nephropathy,” “acute kidney injury,” “chronic kidney disease,” “inflammation,” all of which necessitate further investigation.

**Conclusion:** This study presents a comprehensive bibliometric analysis and offers an up-to-date outlook on the research related to the gut-kidney axis, with a specific emphasis on the present state of intercommunication between gut microbiota and kidney diseases in this field. This perspective may assist researchers in selecting appropriate journals and partners, and help to gain a deeper understanding of the field’s hotspots and frontiers, thereby promoting future research.

## 1 Introduction

According to the Chronic Kidney Disease Collaboration, the global prevalence of chronic kidney disease (CKD) is estimated at 9.1% (GBD Chronic Kidney Disease Collaboration, 2020). With the increasing aging population, and the rising incidence of diabetes and hypertension, the annual incidence of CKD is continuously increasing worldwide. CKD can have various etiologies, in addition to common primary glomerulonephritis (e.g., IgA nephropathy, membranous nephropathy), diabetic kidney disease (DKD) has become another major cause of end-stage kidney disease (ESRD), which significantly affects the patients quality of life and causes substantial socioeconomic burden on society (Coresh, 2017). Moreover, ESRD leads to elevated levels of uremic toxins, which is a major cause of mortality.

The gut microbiota is a complex ecosystem comprised mostly of bacteria, and it constitutes the largest symbiotic ecosystem in the human body (Milani et al., 2017). Its primary physiological function is to preserve the integrity and functionality of the gut, and to regulate immunity, inflammation, and metabolism (Rooks and Garrett, 2016). The gut microbiota has been extensively researched in recent years (Wang et al., 2015; Zhang et al., 2020), especially with the advancement of 16S ribosomal RNA (rRNA) gene sequencing and shotgun metagenomic sequences, resulting in significant changes to this field over the past 2 decades (Huang et al., 2019). Under normal conditions, the host and gut microbiota interact functionally within the gut to achieve mutual benefits, and the composition and abundance of gut microbiota remain relatively stable. However, when the gut microbiota is disturbed beyond the capacity of self-regulation, aberrant modifications can lead to various disorders and multiple diseases, including nervous system diseases, endocrinology disorders, and kidney diseases. In particular, the concept of the gut-kidney axis, introduced by Meijers in 2011, suggests that the gut microbiota can regulate intestine-derived uremic toxins levels to affect CKD progression (Meijers and Evenepoel, 2011). This concept has since been further elaborated on by Meijers in 2019, which highlights the bidirectional communication between gut microbiota and kidney disease (Meijers et al., 2019). On one hand, gut microbiota regulate kidney functions such as inflammation, immunity, uremic toxins, and metabolism. On the other hand, CKD can cause abnormal intestinal barrier function and gut microbiota disorder, facilitating the translocation of bacteria and bacterial components such as endotoxin lipopolysaccharide (LPS) to the bloodstream leading to endotoxemia (Foresto-Neto et al., 2021). Notably, accumulating evidence suggests that the gut microbiota plays an important role in the pathogenesis of IgA nephropathy (IgAN), DKD, and CKD (Coppo, 2018; Yang et al., 2018). The gut-kidney axis has become a well-established concept and a potential therapeutic target for kidney diseases, including diet, probiotics, prebiotics, genetic engineering of bacteria, and fecal microbiota transplants (Krukowski et al., 2023).

In the past 2 decades, substantial evidence has supported the gut-kidney axis theory, and several recent reviews have extensively discussed various aspects of this theory. However, these reviews have mostly focused on specific areas, lacking a comprehensive analysis of the current status and future trends of the topic (Muskiet et al., 2014). Bibliometrics, which was first introduced by Pritchard in 1969, is a statistical analysis method that examines existing literature to identify the development trends and research hotspots in a particular field (Ninkov et al., 2022). This approach has been applied to various disciplines, including medical research (Brandt et al., 2019; Oh and Flaherty, 2020; Ahmad and Slots, 2021; Agnusdei and Coluccia, 2022). By utilizing bibliometric analysis of publications, countries, institutions, journals, authors, and their co-occurrence, we can determine important articles, active authors, and institutions in this field. Furthermore, we used author keywords and keyword co-occurrence analysis to identify the foundation, frontier, and hotspots in the field. However, bibliometric analysis has not yet been used to investigate the gut-kidney axis. Thus, our objective is to reveal the current research status and developing trends of the gut-kidney axis by conducting bibliometric analysis. This study aims to provide directions for future research and to promote the better development of the field.

## 2 Materials and methods

### 2.1 Data sources and search strategy

We conducted a comprehensive search on Web of Science Core Collection (WoSCC), retrieving publications from 2003 to 2022 (up to 30 December 2022) in English language, including both articles and reviews. Both WoSCC and Scopus can be used for bibliometric analysis (Zhang et al., 2017), and we selected WoSCC because it is a collection of high-quality, globally peer-reviewed academic publications that focuses primarily on traditional academic literature, including journal articles, conference proceedings, and books (Falagas et al., 2008). We used the search phrases “(TS = kidney OR TS = renal OR TS = nephropathy OR TS = nephritis OR TS = glomerulonephritis) AND [(TS = gut OR TS = intestinal OR TS = gastrointestinal OR TS = bowel OR TS = fecal) AND (TS = flora OR TS = microbiota OR TS = microbiome OR TS = microbiomes OR TS = microbes OR TS = bacteria OR TS = microflora OR TS = microorganism)]” to retrieve all relevant records. The search timespan covered the years 2003–2022, a period we considered long enough to reflect the development trends in the field we focused on. All relevant records related to the gut-kidney axis were extracted and imported to biblioshiny (using R-Studio) and VOSviewer for bibliometric analysis.

### 2.2 Data analysis

In this study, we utilized the R (version 4.1.2), R-bibliometrix package (version 3.0.3, http://www.bibliometrix.org) and in to analyze the distribution of publication year, country, institutions, journal, authors, and keywords. We used Biblioshiny, a website that offers a web interface for R-bibliometrix, to import data from WoSCC database and conduct visualization analysis of scientific references, including social network maps. Additionally, we used VOSviewer software (version 1.6.16, https://www.vosviewer.com/download), created by Nees Jan van Eck and Ludo Waltman from Leiden University (van Eck and Waltman, 2010), to build maps for co-authorship analysis, keyword co-occurrence analysis. The R package ggplot2 (version 3.4.2) is utilized as a powerful tool for data visualization.

## 3 Results

### 3.1 Evolution of the number of documents and total citations

**FIGURE 1:**
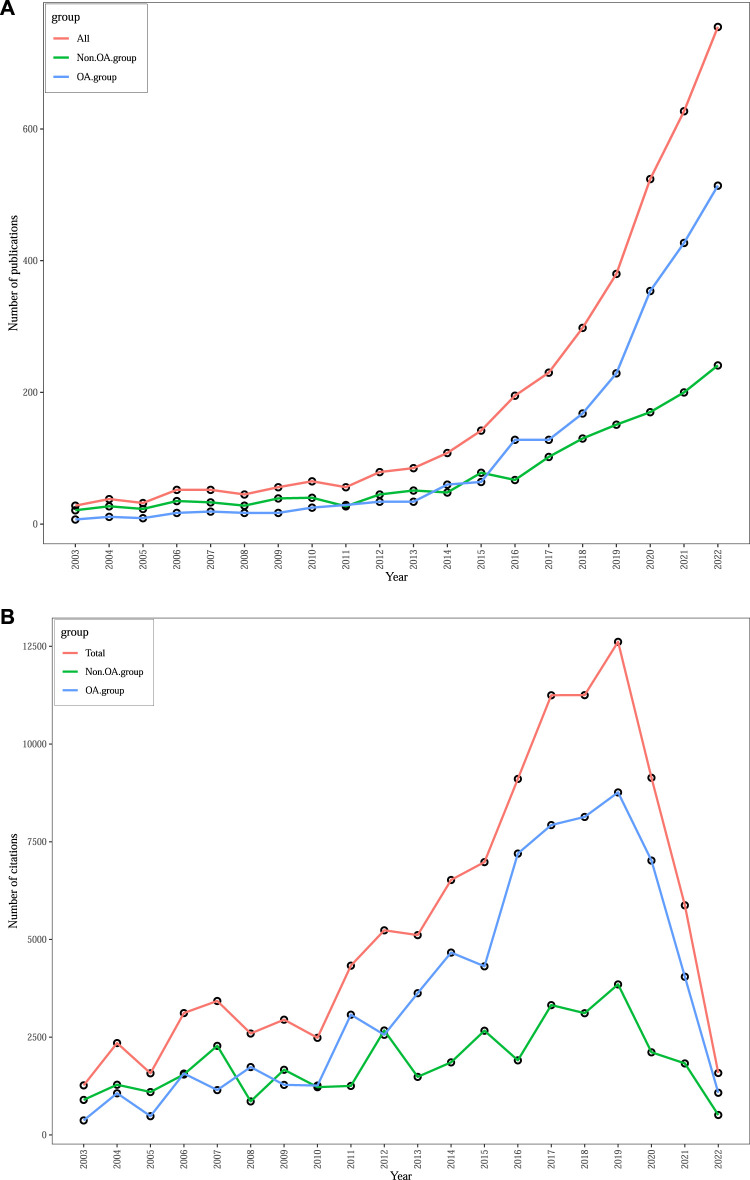
Trends in annual publications **(A)** and total citations **(B)** on gut-kidney axis by using biblioshiny.

The WoSCC database contained a total of 3,900 productions, consisting of 2,608 articles, 979 reviews, and 313 other documents such as editorials, meetings, book chapters, or letters. A histogram (Figure 1) was created to show the number of publications and total citations on the gut-kidney axis over the last 20 years (2003–2022) and to demonstrate its evolution. The number of publications was consistently low from 2003 to 2011, with fewer than 70 documents published. However, since 2012, the number of publications has significantly increased, with a peak in 2022. A total of 1906 documents were published in 2020–2022, which accounted for nearly 50% of all literature. The data indicate a growing interest and attention of scholars towards research on the gut-kidney axis. We also conducted an subgroup analysis of the publication and citation counts of open access and non-open access articles, as shown in Figure 1. Out of the total 3,900 articles, 2,307 (59.15%) were identified as open access, while 1,593 (40.85%) were non-open access. We observed that the growth rate of open access articles (25.37%) exceeded that of non-open access articles (13.7%). Furthermore, Out of a total of 108,845 citations, 71,366 were open access, 37,479 were non-open access, the citations counts indicated that non-open access articles consistently had lower citation counts, with some years showing a declining trend. In contrast, open access articles exhibited a significant increase in citation counts.

**FIGURE 2:**
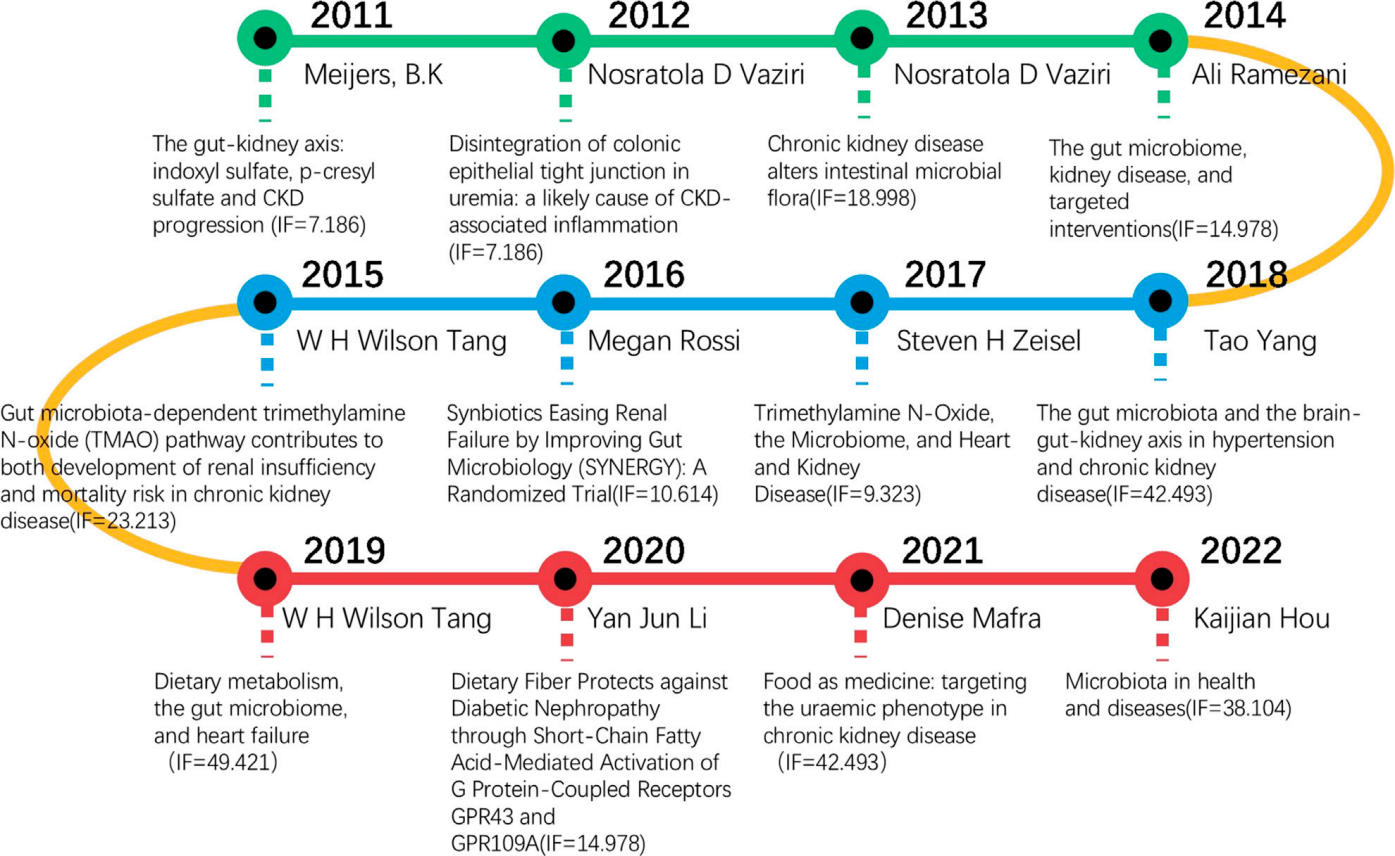
Chronological timeline of key articles in Gut-Kidney Axis research.

The most cited article in WoSCC database belongs to Rui Wang and was published in Phisiological reviews in 2012, under the title “Physiological implications of hydrogen sulfide: a whiff exploration that blossomed,” with 1,338 total citations in WoSCC database. This was followed by a brief report of Jonathan J Havel and other research articles, and the top 20 most cited articles as shown in Table 1. Since 2011, we have systematically identified and selected a series of significant articles through an assessment of the TC count and the relevance of their topics to the gut-kidney axis, aiming to comprehensively understand the progress of gut-kidney axis research. Subsequently, we constructed a chronological timeline to showcase the key milestones represented by these important publications, as shown in Figure 2. The selection of these articles was based on their importance and academic influence within the field of gut-kidney axis research.

**TABLE 1:** Top 20 most cited articles.

Title	Author	Journal	year	Total citations	Impact factor	JCR Partition
Physiological implications of hydrogen sulfide: a whiff exploration that blossomed	Rui Wang	PHYSIOL REV	2012	1,338	46.5	Q1
The evolving landscape of biomarkers for checkpoint inhibitor immunotherapy	Jonathan J Havel	NAT REV CANCER	2019	1,128	69.8	Q1
Inflammageing: chronic inflammation in ageing, cardiovascular disease, and frailty	Luigi Ferrucci	NAT REV CARDIOL	2018	999	49.421	Q1
Gut Microbiota in Cardiovascular Health and Disease	W H Wilson Tang	CIRC RES	2017	753	23.213	Q1
Gut microbiota-dependent trimethylamine N-oxide (TMAO) pathway contributes to both development of renal insufficiency and mortality risk in chronic kidney disease	W H Wilson Tang	CIRC RES	2015	693	23.213	Q1
Olfactory receptor responding to gut microbiota-derived signals plays a role in renin secretion and blood pressure regulation	Jennifer L Pluznick	P NATL ACAD SCI USA	2013	654	12.779	Q1
Chronic kidney disease alters intestinal microbial flora	Nosratola D Vaziri	KIDNEY INT	2013	652	18.998	Q1
Non-alcoholic fatty liver disease and its relationship with cardiovascular disease and other extrahepatic diseases	Leon A Adams	GUT	2017	571	31.793	Q1
Bacterial infections in cirrhosis: a position statement based on the EASL Special Conference 2013	Jonathan R Swann	J HEPATOL	2014	534	30.083	Q1
Systemic gut microbial modulation of bile acid metabolism in host tissue compartments	Jonathan R Swann	P NATL ACAD SCI USA	2011	474	12.779	Q1
High-Fiber Diet and Acetate Supplementation Change the Gut Microbiota and Prevent the Development of Hypertension and Heart Failure in Hypertensive Mice	Francine Z Marques	CIRCULATION	2017	473	39.918	Q1
Negative association of antibiotics on clinical activity of immune checkpoint inhibitors in patients with advanced renal cell and non-small-cell lung cancer	L Derosa	ANN ONCOL	2018	446	51.769	Q1
Antigen-independent differentiation and maintenance of effector-like resident memory T cells in tissues	Kerry A Casey	J IMMUNOL.	2012	427	5.462	Q1
The gut microbiome, kidney disease, and targeted interventions	Ali Ramezani	J AM SOC NEPHROL	2014	420	14.978	Q1
Metabolic footprint of diabetes: a multiplatform metabolomics study in an epidemiological setting	Karsten Suhre	PLOS ONE	2010	420	3.752	Q2
Clinical pharmacokinetics and pharmacodynamics of mycophenolate in solid organ transplant recipients	Christine E Staatz	CLIN PHARMACOKINET	2007	416	5.577	Q1
Obesity and cancer risk: Emerging biological mechanisms and perspectives	Konstantinos I Avgerinos	METABOLISM	2019	415	13.934	Q1
Prognostic value of elevated levels of intestinal microbe-generated metabolite trimethylamine-N-oxide in patients with heart failure: refining the gut hypothesis	W H Wilson Tang	J AM COLL CARDIOL	2014	413	27.203	Q1
Risk of *Clostridium difficile* diarrhea among hospital inpatients prescribed proton pump inhibitors: cohort and case-control studies	Sandra Dial	CAN MED ASSOC J	2004	410	16.859	Q1

### 3.2 Journal analysis

**FIGURE 3:**
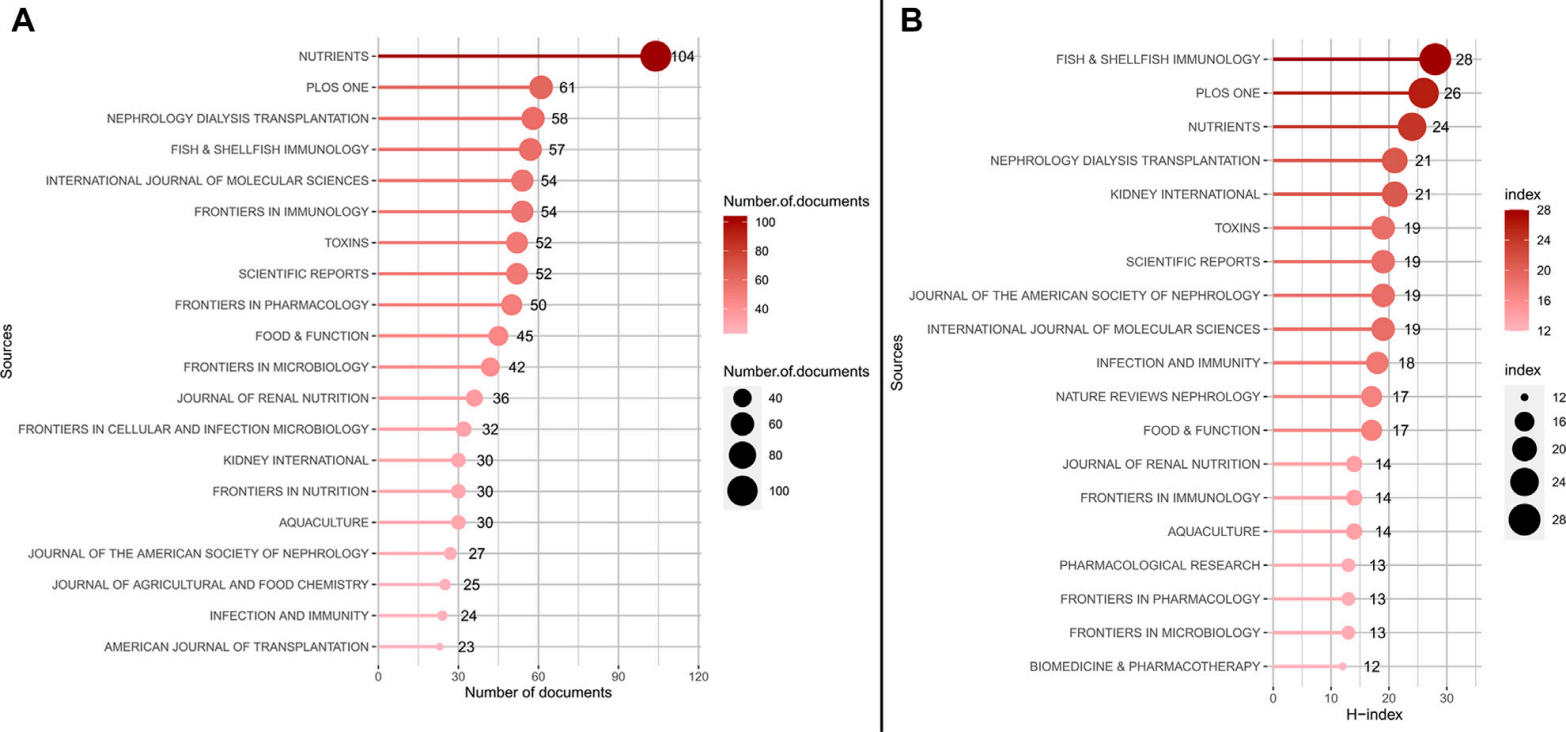
The most productivity and influential in research on the gut-kidney axis by using biblioshiny. **(A)** Top 20 most productive journals contributing to research. **(B)** Top 20 journals impact ranked by H-index.

The most productivity and influential in research on the gut-kidney axis by using biblioshiny were shown in Figure 3. Figure 3A displays the top 20 journals that published articles on the gut-kidney axis between 2003–2022. Out of the 1,225 journals that were identified, 602 journals were non-open access journals, while 517 journals were open access journals. 22.71% of the total articles were published in the top 20 journals, totaling 886 articles. Nutrients, with an impact factor of 6.71 and a JCR (Journal Citation Reports) Q1, published the most articles, accounting for 2.67% of all publications, followed by PLoS One (1.56%), Nephrology Dialysis Transplantation (1.49%), Fish & Shellfish Immunology (1.46%), Frontiers in Immunology (1.38%), International Journal of Molecular Sciences (1.38%), Scientific Reports (1.33%), and Toxins (1.33%).

The H-Index is a measure used to evaluate the number and impact of journals (Hirsch, 2005). According to Figure 3B and Table 2, the most influential journals based on H-Index are Fish and Shellfish Immunology (H-Index = 28), PLoS One (H-Index = 26), and Nutrients (H-Index = 24). It is worth noting that although Fish and Shellfish Immunology has only published 57 articles, it has an H-Index of 28, which is the highest among all journals. In addition, two nephrology journals, Kidney International and Nephrology Dialysis Transplantation, have an H-Index of 21, ranking fourth.

**TABLE 2:** Top 20 most productive journals.

Rank	Journals	Documents	Impact factor	JCR Partition	H-index
1	Nutrients	104	6.706	Q1	28
2	PLoS One	61	3.752	Q2	26
3	Nephrology Dialysis Transplantation	58	7.186	Q1	24
4	Fish & Shellfish Immunology	57	4.622	Q1	21
5	Frontiers in Immunology	54	8.786	Q1	21
6	International Journal of Molecular Sciences	54	6.208	Q1	19
7	Scientific Reports	52	5.000	Q1	19
8	Toxins	52	5.075	Q1	19
9	Frontiers in Pharmacology	50	5.988	Q1	18
10	Food & Function	45	6.317	Q1	18
11	Frontiers in Microbiology	42	6.064	Q1	16
12	Journal of Renal Nutrition	36	4.354	Q2	15
13	Frontiers in Cellular and Infection Microbiology	32	6.073	Q1	15
14	Aquaculture	30	5.135	Q1	14
15	Frontiers in Nutrition	30	6.590	Q1	14
16	Kidney International	30	18.988	Q1	13
17	Journal of the American Society of Nephrology	27	14.980	Q1	13
18	Journal of Agricultural and Food Chemistry	25	5.890	Q1	12
19	Infection and Immunity	24	3.610	Q3	12
20	American Journal of Transplantation	23	9.370	Q1	12

**FIGURE 4:**
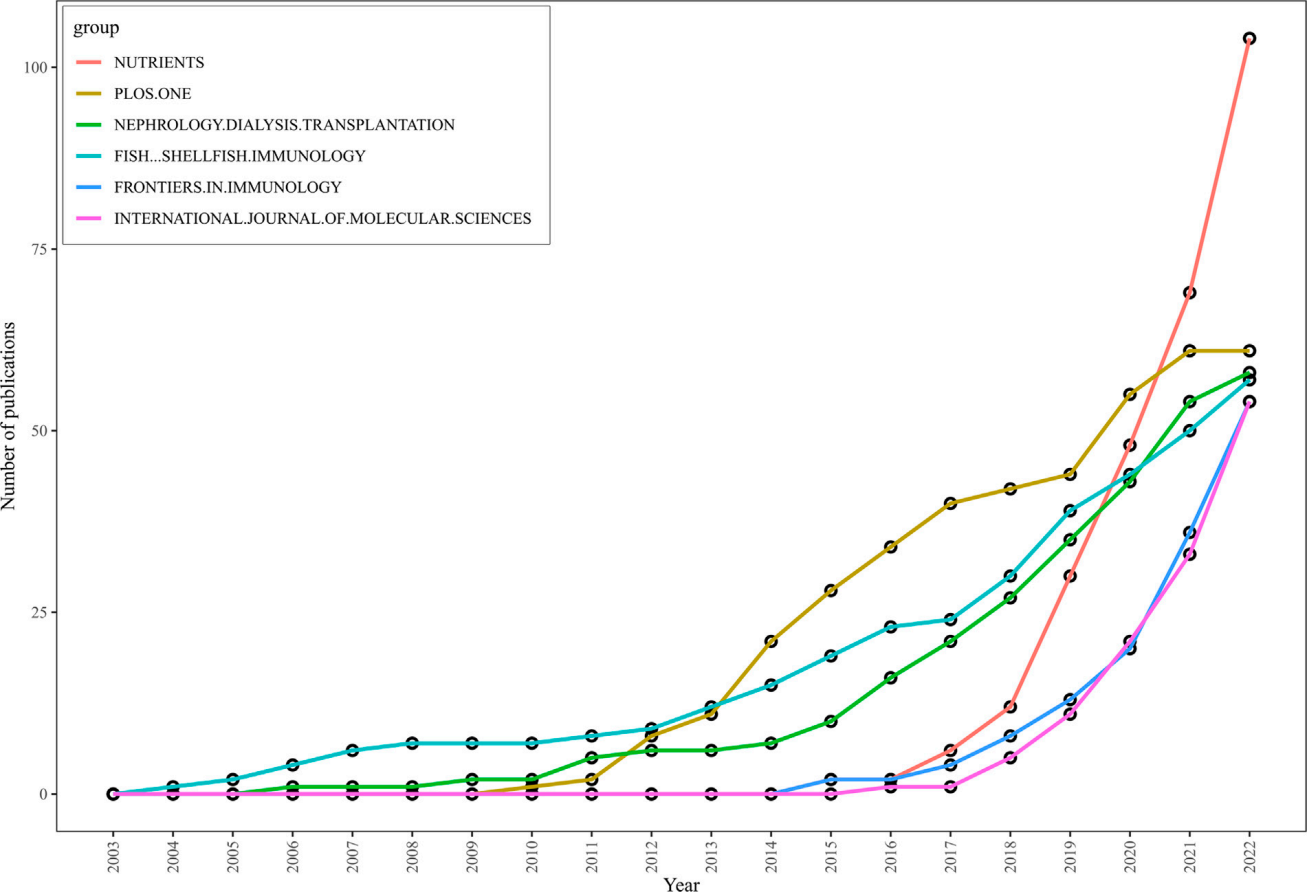
Top 6 journals publications trends overtime by using biblioshiny.

Based on the growth curve of the top six journals (shown in Figure 4), PLoS One has consistently been a popular journal in this field, but Nutrients has experienced the fastest growth in recent years. As of 2021, the total number of documents in Nutrients exceeded that of PLoS One and is still increasing, indicating that modern research has discovered a close link between gut microbiota, metabolism, and nutrition, and research in this area is on the rise.

### 3.3 Active countries and institutions

**FIGURE 5:**
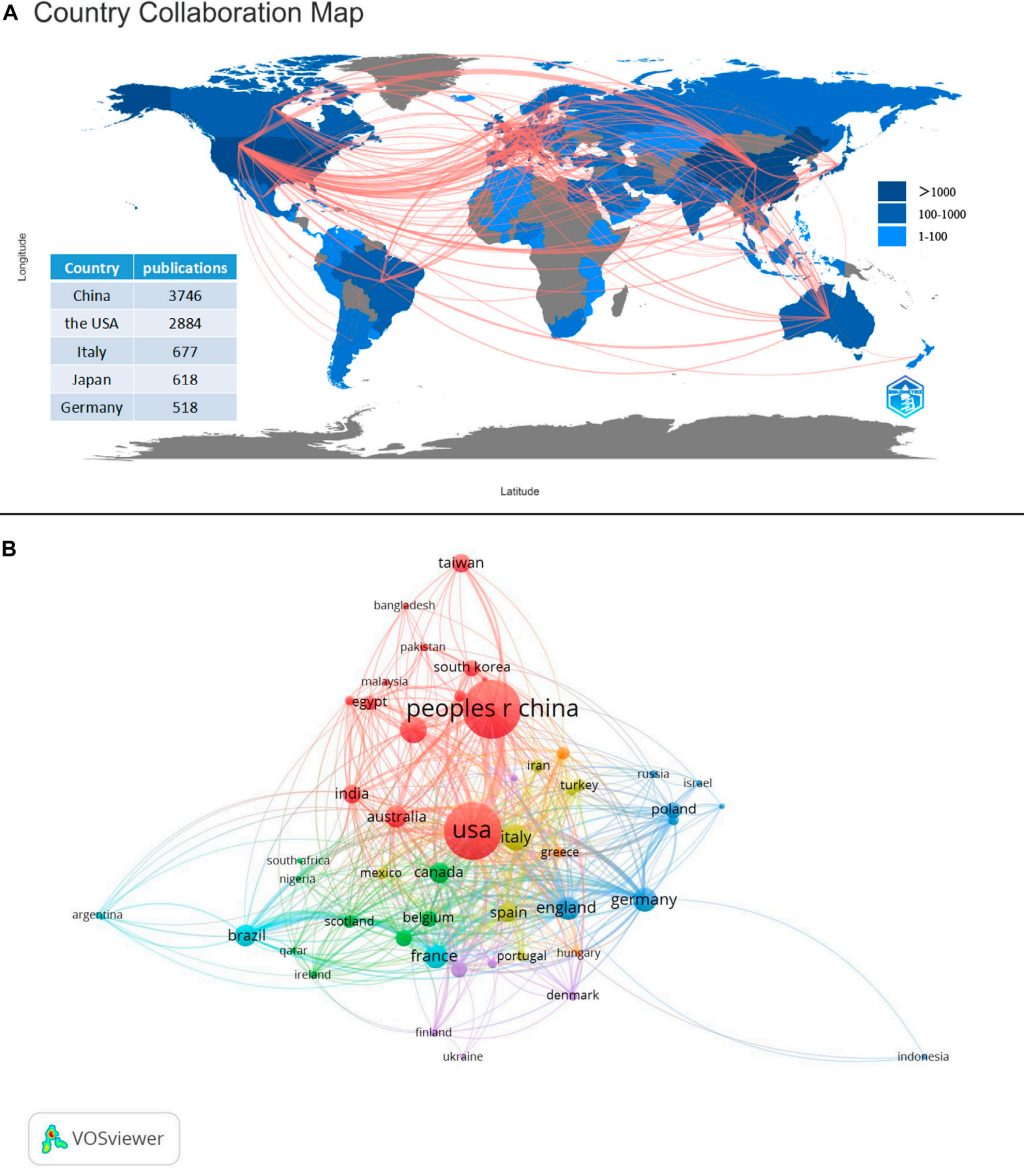
Countries contribution and collaboration. **(A)** Countries contribution of gut-kidney axis based on total publications and collaboration between contributing countries by using biblioshiny. **(B)** Countries collaboration map with a minimum number of documents of a country more than 10 by using vosviewer.

**FIGURE 6:**
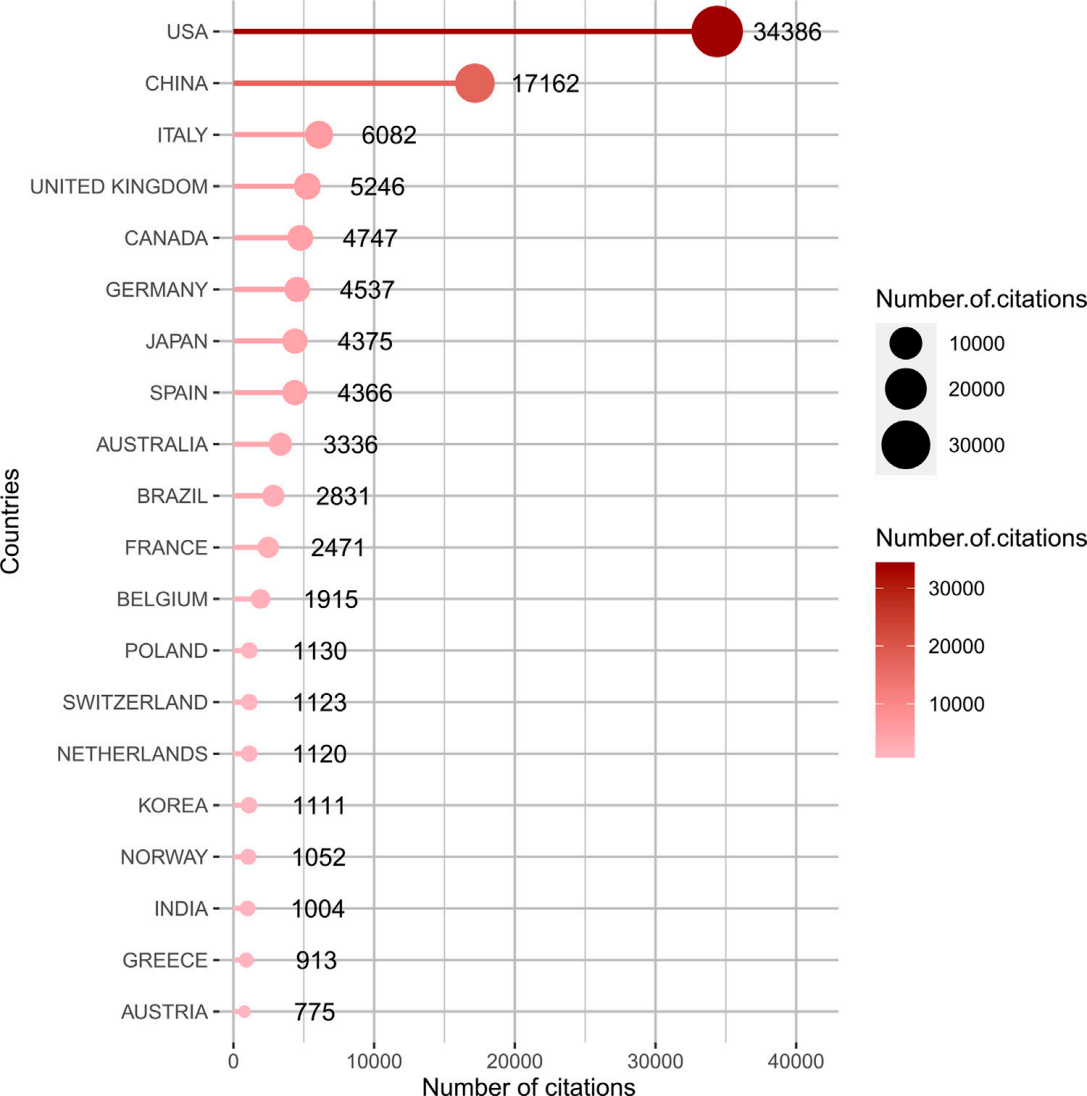
Top 20 Most cited countries by using biblioshiny.

The number of publications and a collaboration map for 94 countries involved in research on the gut-kidney axis was presented by using biblioshiny (Figure 5A) and vosviewer (Figure 5B). China was the most productive country with 3,746 articles, followed by the United States with 2,884, Italy with 677, Japan with 648, and Germany with 518. The United States and China were responsible for the majority of the research on this topic, and the number of citations reflects the countries influence in the field (Figure 6). The United States had over 30,000 citations, while China had 17,136, indicating that the United States maintained a leading position. Both countries had a significant increase in publications, especially the United States in 2020. However, the impact of Chinese papers could be improved as they had a low citation frequency of 4.57. China is making progress in the gut-kidney axis field, and it could increase its influence by collaborating more closely with other countries and producing innovative research. Figure 5 also displays a map of country collaborations among the top productive countries. Connected lines between two countries indicate their level of cooperation, which is determined by the line’s thickness and quantity. The United States, China, and Western European countries demonstrate strong collaboration and exchange. Out of the 93 countries involved in international cooperation, the United States has the highest number of international research collaborations with other countries.

**FIGURE 7:**
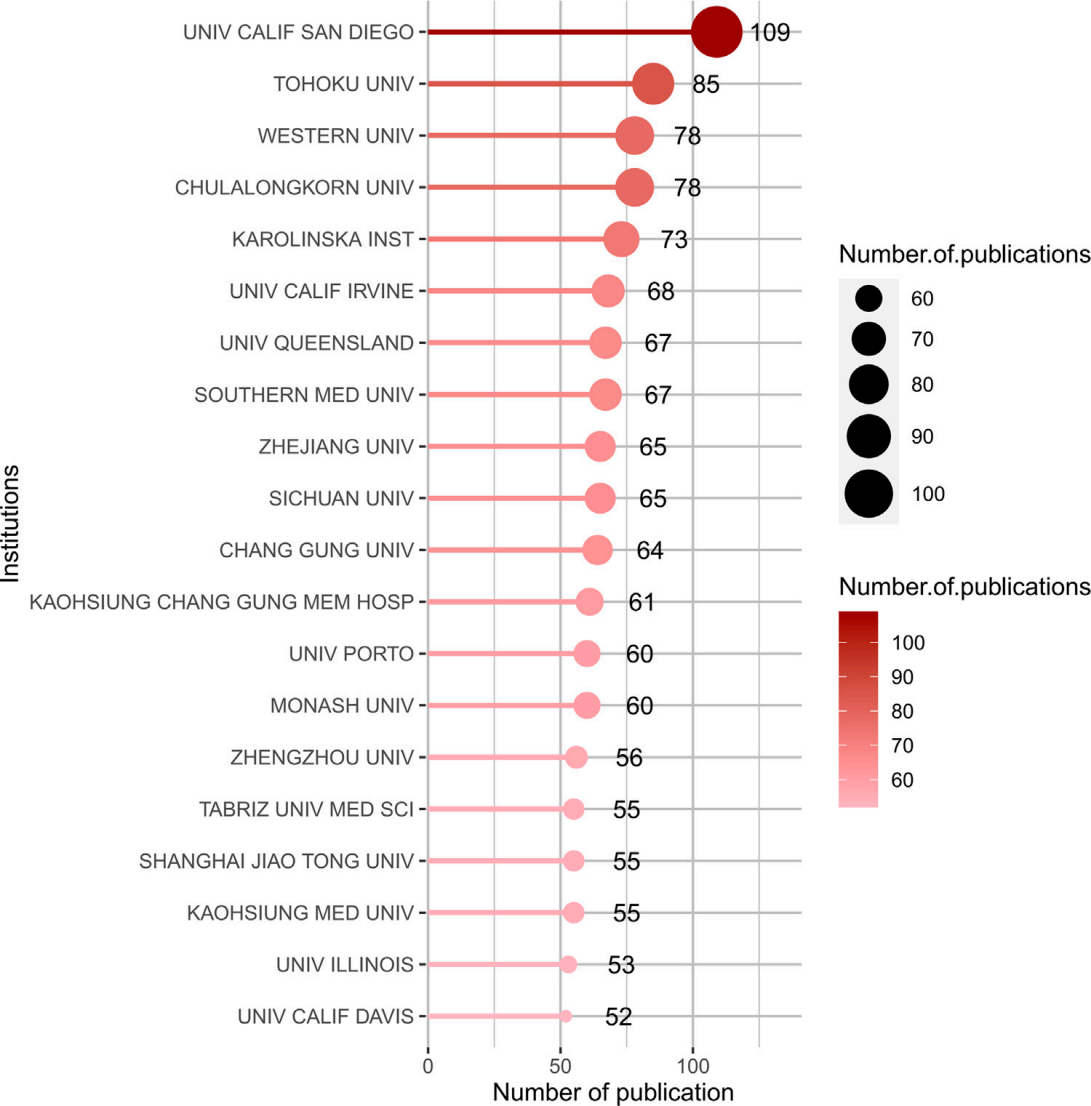
Top 20 productive institutions concerning the research of gut-kidney axis by using biblioshiny.

The publications were contributed by 4,150 institutions worldwide, top 20 productive institutions concerning the research of gut-kidney axis by using biblioshiny was shown in Figure 7 with 70% (14) of the top 20 institutions located in the United States (6, 30%) and China (8, 40%). The remaining 6 (35%) of the top 20 institutions are located in Japan (1), Thailand (1), Sweden (1), Malaysia (1), Portugal (1), and Iran (1), as shown in Figure 6. The University of California San Diego had the highest number of published articles with 109 articles, followed by Tohoku University (85 publications), Chulalongkorn University (78 publications), Western University (71 publications), Karolinska Institutet (69 publications), University of California--Irvine (68 publications), The University of Queensland (66 publications) Sichuan University (65 publications) and Chang Gung University (64 publications).

### 3.4 Authors analysis

**FIGURE 8:**
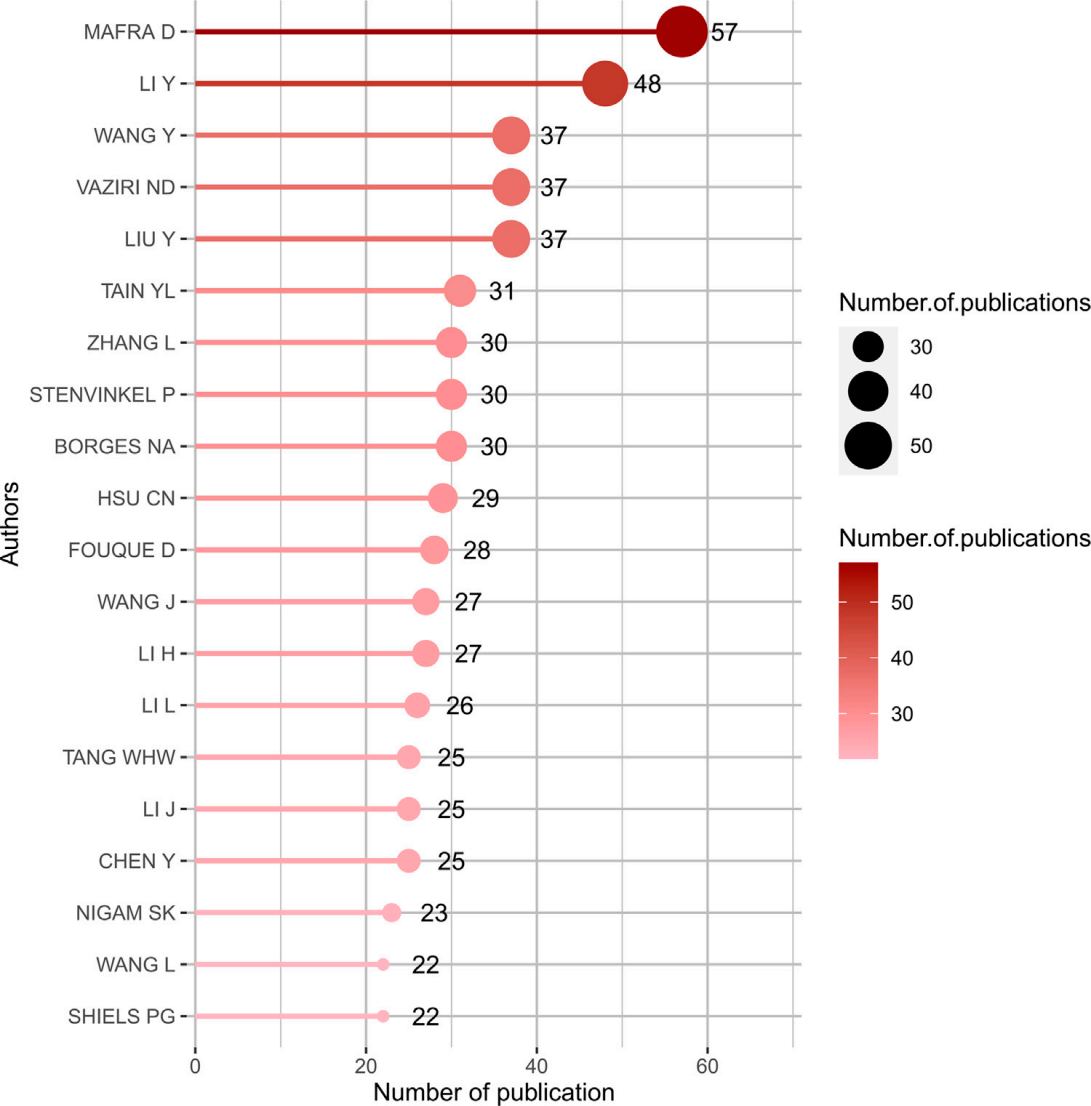
Top 20 productive authors on gut-kidney axis by using biblioshiny.

**FIGURE 9:**
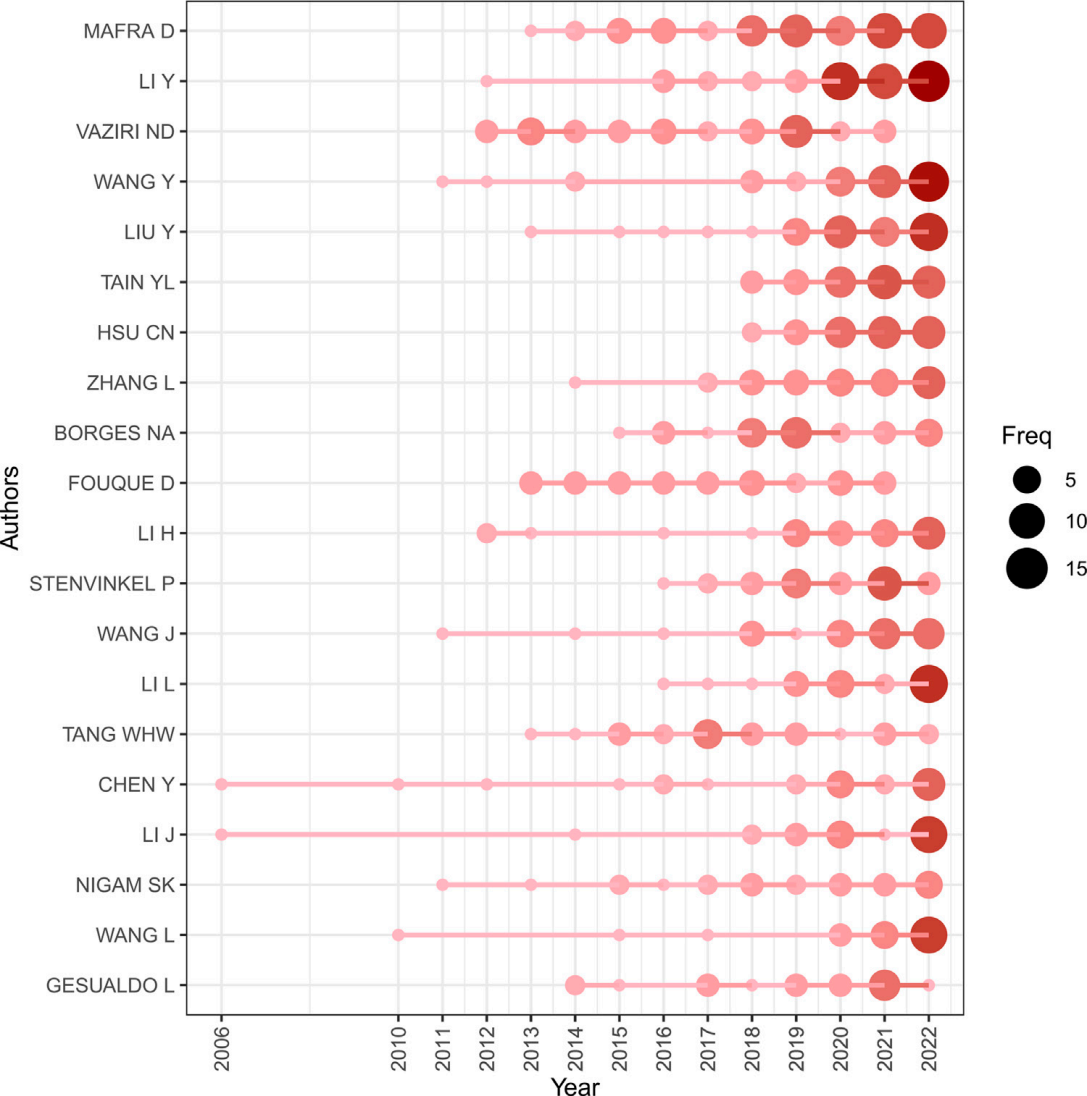
Top 20 author’s production over time by using biblioshiny.

**FIGURE 10:**
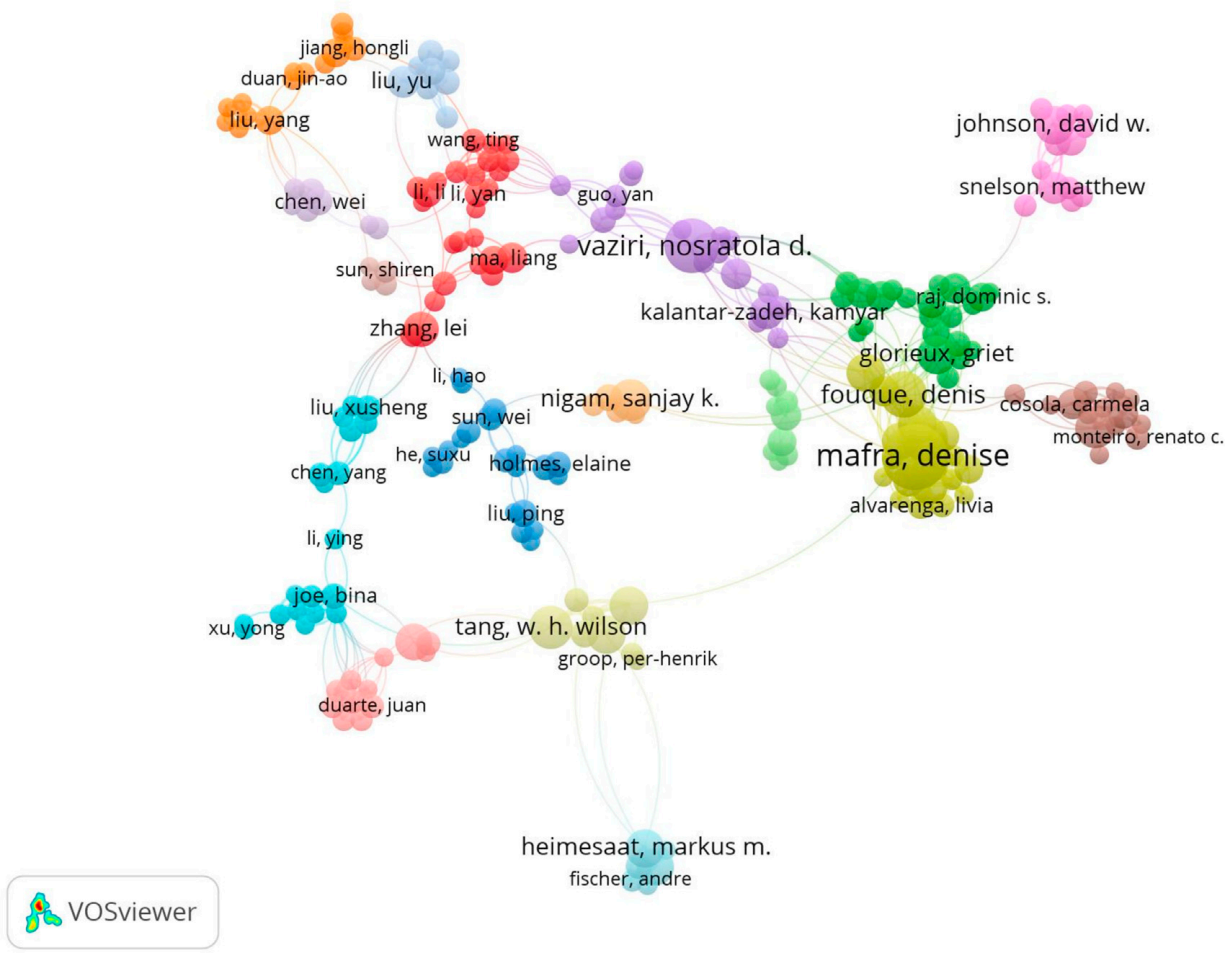
The visualization map author’s network by using VOSviewer.

A total of 17,943 authors were involved in the publication of documents related to the gut-kidney axis, with an average of 4.60 authors per article. The top 20 authors with the largest output published 616 articles (Figure 8), accounting for 15.79% of all publications. Denise Mafra from the Federal University of Rio de Janeiro, Brazil, had the highest number of publications with 57 articles. In 2021, an article on the effect of food as a drug on CKD was published in Nat Rev Nephrol, which explained the impact of food on intestinal flora and had 29 citations. Li Y (48 articles), Liu Y (37 articles), and Nosratola D Vaziri (37 articles) followed closely behind. Based on the publication output of the top 20 authors over time (Figure 9), most authors published articles every year, indicating that they maintain their research interest in the field. Li Y’s research has spanned a long period, with articles published in the field since 2004. Most authors gained a strong interest in the field since 2011, when the concept of the gut-kidney axis was proposed by Meijers. To identify core authors and explore the cooperative relationships among authors, a collaboration network visualization was performed by VOSviewer, as presented in Figure 10. For better visualization, the author collaboration network only describes 347 authors with at least 5 papers. The collaboration between authors was close, while the centrality of each author was much less than 0.1. Denise Mafra had the strongest cooperation with others, with a total link strength of 180, followed by Stenvinkel Peter (86) and Tain You-lin (83).

### 3.5 Keyword analysis and keyword co-occurrence analysis

**FIGURE 11:**
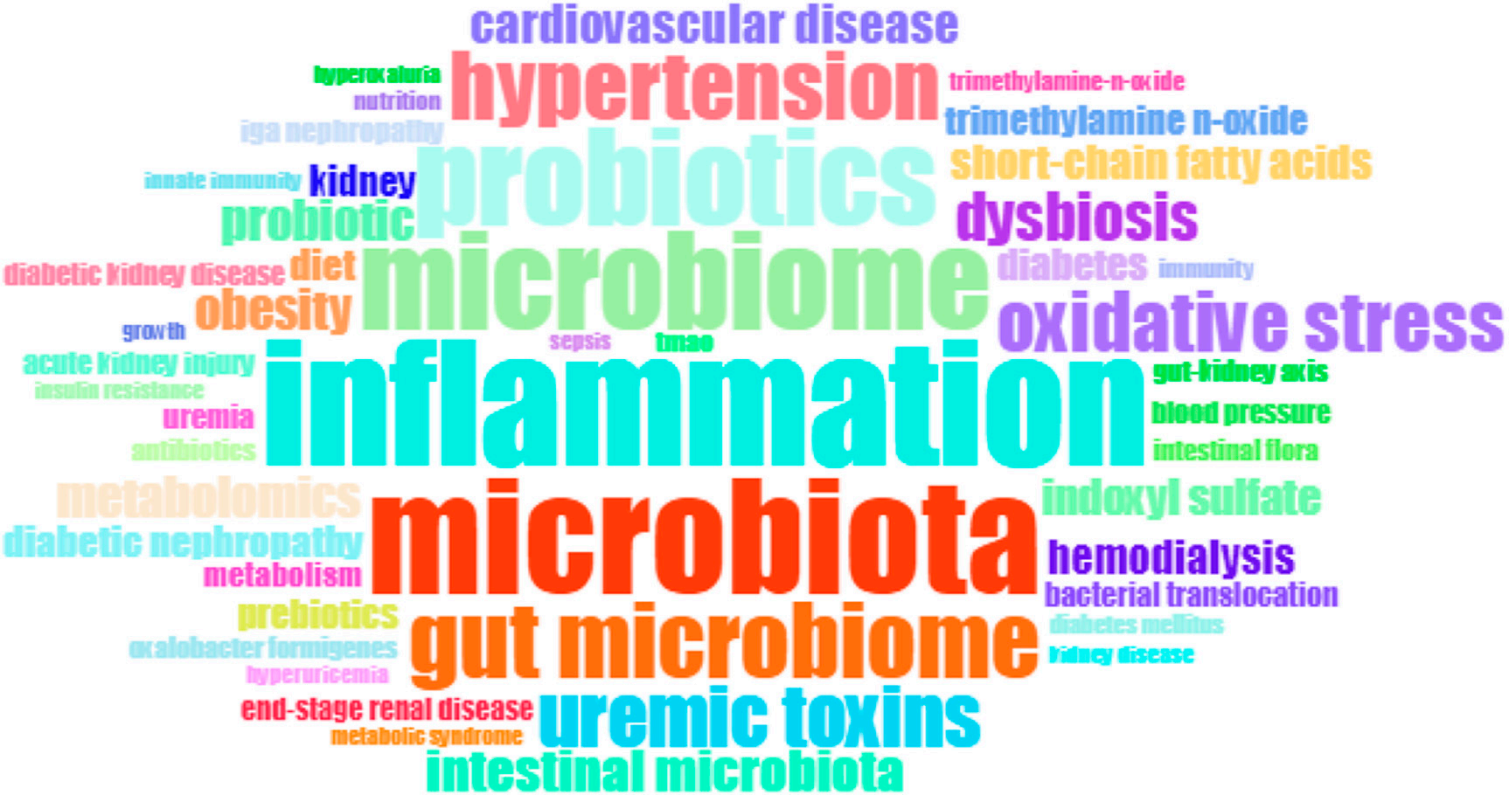
Word cloud based on author’s keyword by using biblioshiny.

**FIGURE 12:**
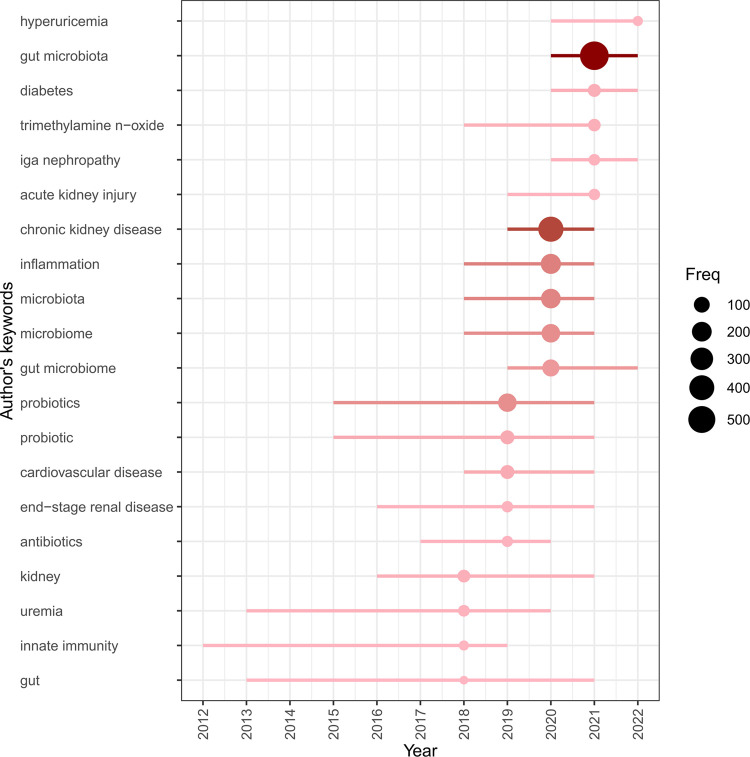
Trend topics based on author’s keyword over time by using biblioshiny.

**FIGURE 13:**
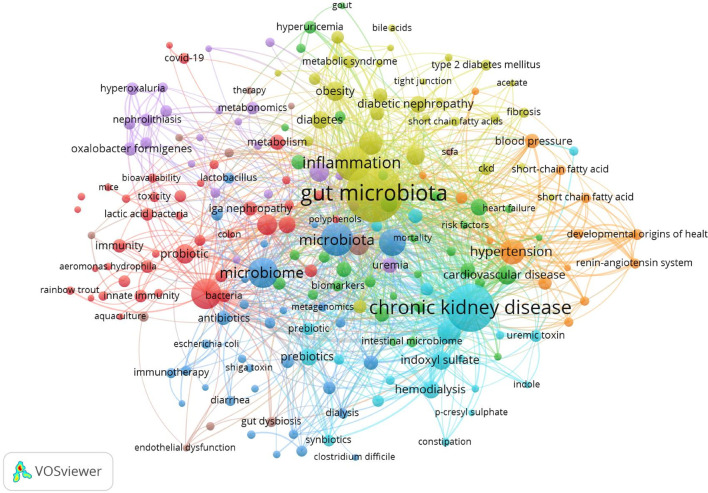
The overlay co-occurrence visualization network of 229 keywords with a frequency of more than 10 times for gut-kidney axis by using VOSviewer.

A total of 7,348 keywords were identified in the 3,900 gut-kidney axis-related documents using the biblioshiny app’s “Author’s keywords” and Thomson Reuters editorial expertize team’s “Keywords Plus” for thematic trend analysis. However, the comparison between the two revealed that “Author’s keywords” were more precise and thus used as the primary source of analytical data. A Word Cloud map was used to represent the number of occurrences of each keyword, as presented in Figure 11. The term “gut microbiota” was the most frequently occurring keyword with 590 mentions, followed by “chronic kidney disease” with 411 mentions and “inflammation” with 212 mentions. The trend over time showed that the high-frequency keywords in recent years were “hyperuricemia,” “gut microbiota,” “diabetes,” “trimethylamine n-oxide,” “iga nephropathy,” “acute kidney injury,” “chronic kidney disease,” “inflammation” and etc. (Figure 12). The VOSviewer software was used to analyze the keywords in the 3,900 articles. Keyword co-occurrence analysis was conducted to investigate the relationships between keywords and identify hot topics. This approach can help researchers gain a better understanding of scientific findings in research hotspots. Using the “Author’s Keywords” parameter, a keyword co-occurrence network was established among the 204 main keywords filter with 10 minimum occurrences (Figure 13), and four clusters with different colors (yellow, red, blue, and green) were mainly identified. Nodes in the same cluster with the same colors represented close co-occurring relationships, and the node size and link line width were proportional to the extent and strength of co-occurrence. The betweenness centrality was used to measure the importance of nodes in the network, with the larger and higher total link strength nodes being more significant, indicating that more information passed through them. The largest and highest total link strength node was the “gut microbiota” in the yellow cluster, which was a prominent hot topic. The above keywords were mainly divided into the following four clusters through keyword co-occurrence analysis, as shown in Table 3. By conducting a thorough analysis of these four clusters, we can observe that each clusters is associated with a certain disease. The yellow cluster encompasses terms related to kidney diseases associated with metabolism, such as diabetes, obesity, and diabetic nephropathy. The red clusters place more emphasis on the immune system and bacteria infection in the interaction between the gut and the kidney, these topics are more frequently studied in relation to IgAN. The blue cluster and green cluster, on the other hand, is focused more on urinary toxins, such as indoxyl sulfate, p-cresyl sulfate, and trimethylamine-n-oxide which is related to CKD and cardiovascular disease.

**TABLE 3:** Four main cluster of author keyword ranked by occurrences.

Rank	Yellow cluster	Red cluster	Blue cluster	Green cluster
1	inflammation	probiotics	chronic kidney disease	cardiovascular disease
2	oxidative stress	intestinal microbiota	uremic toxins	diet
3	obesity	probiotic	indoxyl sulfate	trimethylamine n-oxide
4	short-chain fatty acids	kidney	hemodialysis	bacterial translocation
5	diabetes	metabolism	prebiotics	acute kidney injury
6	diabetic nephropathy	iga nephropathy	gut-kidney axis	tmao
7	diabetic kidney disease	immunity	kidney disease	intestinal flora
8	diabetes mellitus	innate immunity	prebiotic	hyperuricemia
9	nutrition	growth	p-cresyl sulfate	sepsis
10	insulin resistance	bacteria	synbiotics	intestinal barrier
11	metabolic syndrome	gut	uremic toxin	metabolites
12	ckd	lactic acid bacteria	metabolome	biomarkers
13	type 2 diabetes	immune response	symbiotic	gastrointestinal microbiome
14	type 2 diabetes mellitus	infection	renal fibrosis	endotoxin
15	apoptosis	pharmacokinetics	p-cresol	lipopolysaccharide
16	fibrosis	COVID-19	constipation	gastrointestinal tract
17	dietary fiber	cytokines	vascular calcification	intestinal microbiome
18	short chain fatty acids	toxicity	chronic kidney disease	cardiovascular diseases
19	autophagy	liver	urea	choline
20	bile acids	antioxidant	indole	cirrhosis

## 4 Discussion

### 4.1 Summary of findings

In recent years, there has been a growing interest in the role of the gut microbiota in the pathogenesis of kidney disease, making the gut-kidney axis a hot topic in the field of kidney diseases. This study represents the first attempt in the literature to apply bibliometric analysis to the study of the gut-kidney axis. Using R software and VOSviewer, we conducted a bibliometric visualization analysis of 3,900 publications related to the gut-kidney axis in the WoSCC database. The findings of this study provide a comprehensive review of the research hotspots related to the gut-kidney axis over the past 2 decades.

Based on publication trends, there has been a significant increase in the number of publications related to the gut-kidney axis since 2011, which may be attributed to the emergence of the concept. Moreover, numerous researchers have extensively investigated the crosstalk between gut microbiota and kidney diseases, and such research is ongoing. What’s more, our findings highlight the positive impact of open access publishing in the gut-kidney axis field. Open access articles not only demonstrate higher publication growth rates but also attract more citations, indicating their broader impact and potential for collaboration and knowledge dissemination. These results underscore the importance of data availability and accessibility in facilitating scientific progress and advancing research within the gut-kidney axis field.

An analysis of journal publications has shown that the number of publications in the field of nutrition and metabolism, as covered by Nutrient, has been rapidly increasing, indicating that these topics are currently research hotspots. The United States and China have been leading countries in this field, with China, as a developing country, striving to catch up in scientific research. While there have been more scientific productions in the United States than in China, the overall quality of articles in China is lower, resulting in a low academic influence worldwide. The United States has a significant advantage over other countries in terms of academic influence, as evidenced by the number of citations. Furthermore, the United States has engaged in extensive cooperation with China, Japan, and various European countries. Among the top 20 research institutions, the most active is the University of California, San Diego in the United States, followed by Tohoku University in Japan, while several Chinese institutions also rank highly. However, the low average number of publications among Chinese institutions reveals a gap between these institutions and those in other countries.

According to author analysis, Denise Mafra is the most prolific author and has extensive collaborations with other authors, with a focus on the impact of diet on gut microflora and kidney disease. Mafra promotes the theory of “Food is Medicine” and recently published an article in Nature Reviews Nephrology emphasizing the crucial role of diet in modulating the gut microbiota and affecting the health status of CKD patients (Mafra et al., 2021). Vaziri ND, who closely collaborates with Mafra, contributes greatly to the field and specializes in abnormal lipid metabolism in kidney disease, recognizing the importance of gut microbiota in regulating metabolic disorders (Vaziri et al., 2010; Vaziri et al., 2013; Vaziri, 2016). Many Chinese authors, such as Ma Liang, Li Yan, and Wang ting, have strong collaborations with Mafra and Vaziri ND, forming a red cluster in the cooperation network. Meanwhile, another cluster is centered on Tang, W. H. Wilson, who focuses on the role of gut microbiota in cardiovascular disease and cardiorenal syndrome (Tang et al., 2017; Tang et al., 2019), although he is not the most prolific author in this field. Other researchers, such as Johnson DW, Matthew Snelson, in the purple cluster, their collaborations shows the strong association between gut microbiota and CKD (McFarlane et al., 2021; McFarlane et al., 2022).

**FIGURE 14:**
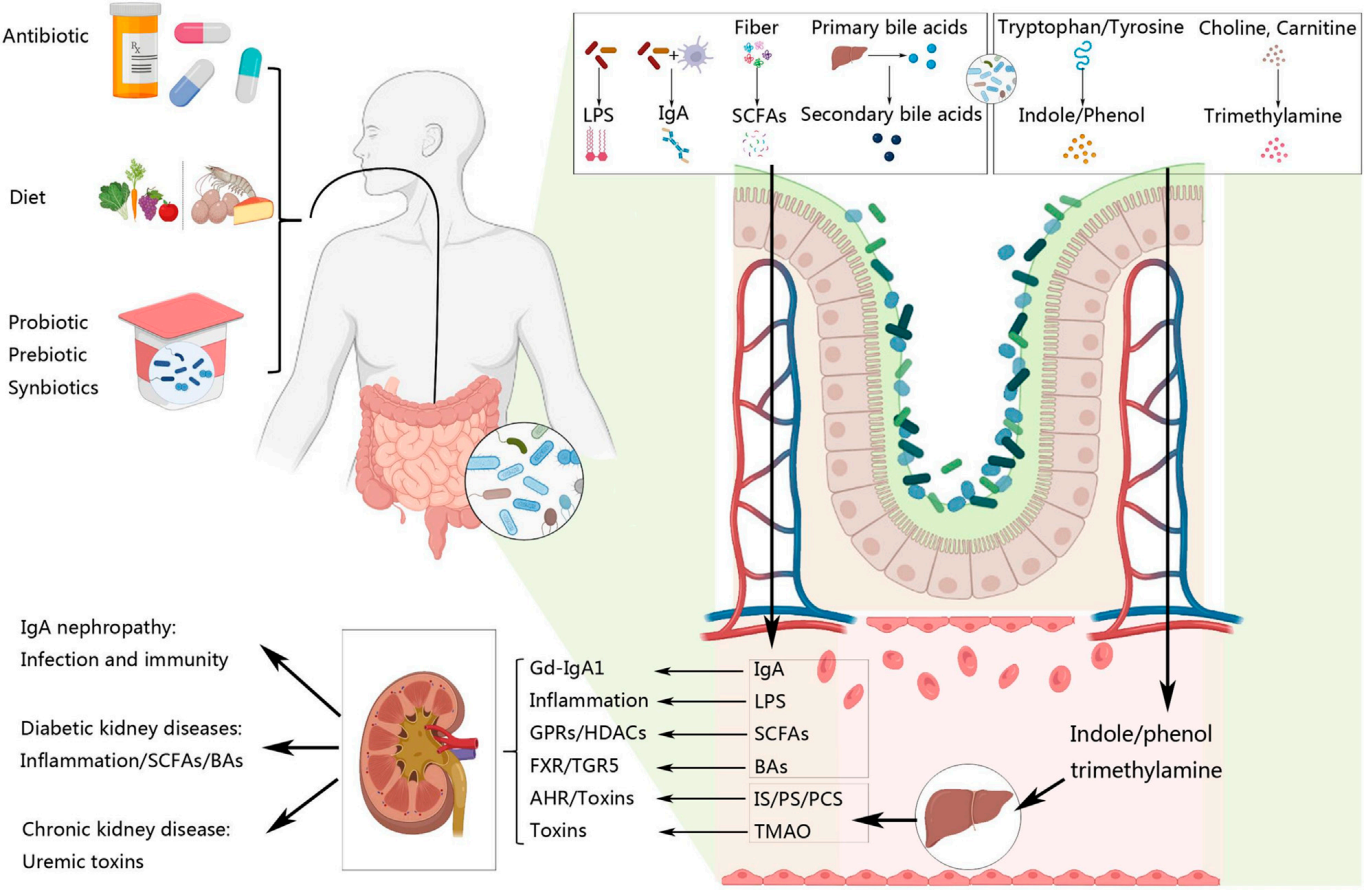
Interaction between gut microbiota and kidney.

Overall, the current research hotspots of the gut-kidney axis mainly focus on the molecular mechanisms underlying the crosstalk between gut microbiota and different diseases related to kidney. Author keyword co-occurrence network analysis categorizes the keywords into four main clusters, focusing on immunity, inflammation, metabolism, urine toxins. And we will elaborate along these mechanisms of the gut-kidney axis in this paper (Figure 14).

### 4.2 Diabetic nephropathy: inflammation, short-chain fatty acids and bile acids

Dysbiosis of the gut microbiota and chronic intestinal inflammation are closely related to the occurrence of various inflammatory kidney diseases. The intestinal barrier function refers to the well-developed functional barrier in the normal intestine, which separates the gut lumen from the host environment and prevents the entry of pathogenic antigens. The intestinal mucosal epithelial cells and intercellular tight junctions, mainly composed of tight junction proteins including occludin, claudin-1, and zonula occludens-1, are responsible for this barrier (Lee et al., 2018). Factors that disrupt the gut ecology can also lead to the disruption of the intestinal tight junctions, or “leaky gut” (Kinashi and Hase, 2021). The increase of harmful bacteria leads to the infiltration of inflammatory cells, followed by a significant reduction in the key protein components of the epithelial tight junction (Chelakkot et al., 2018). In addition, uremic toxins aggravate gut permeability in both uremic rats and CKD patients (Vaziri et al., 2012). “Leaky gut” further allows endotoxins such as LPS to translocate into the bloodstream, which further stimulates Toll-like receptors-4 (TLR-4), inducing the production of pro-inflammatory cytokines such as interleukin-6 (IL-6), interferon-γ (IFN-γ), and tumor necrosis factor-α (TNF-α) via nuclear factor kappa-B (NF-κB), leading to systemic inflammatory reactions (Ghosh et al., 2020). The abundance of probiotics in the gut of CKD patients is decreased, while the levels of inflammatory factors are increased, and research suggests that the levels of these probiotics are negatively correlated with the levels of inflammatory factors (Li et al., 2019). In addition, a significant increase in Gram-negative bacteria is also found in type 2 diabetes and CKD patients, which contain LPS in the outer membrane and are associated with the elevation of inflammatory biomarkers such as TNF-α, IL-6, and C-reactive protein (CRP) in fecal microbiota samples (Salguero et al., 2019).

In recent years, with the deepening of research, it has been discovered that the gut microbiota can affect various pathways of human metabolism. Among them, the metabolites of the gut microbiota can affect the physiological functions of different tissues and organs, thereby regulating the overall metabolism of the body. Through keyword analysis, it has been found that two key metabolites are bile acids and short-chain fatty acids, which are closely related to metabolic kidney diseases such as DKD, obesity-related kidney disease, and hypertension-related kidney disease. Keywords that affect body metabolism include insulin resistance, metabolic syndrome, diet-induced obesity and etc. Short chain fatty acids (SCFAs) are a group of fatty acids with fewer than six carbon atoms, including acetate, propionate, butyrate and et al. (Kimura et al., 2020). They are produced by the bacterial fermentation of dietary fiber and play a key role in maintaining gut health and metabolic homeostasis (Martin-Gallausiaux et al., 2021). SCFAs have been shown to stimulate the release of gut hormones, improve insulin sensitivity, and reduce inflammation. The physiological effects of SCFAs are mainly as ligands of G Protein-Coupled Receptors (GPCRs) or as inhibitors of histone deacetylase (HDAC) (Fawad et al., 2022). Common GPCRs include GPR41, GPR43, and GPR109a (Kim et al., 2013; Li et al., 2020). GPR41 and GPR43 are widely distributed in adipose tissue, pancreatic tissue, and gastrointestinal tissue. Activation of GPR41 and GPR43 by SCFAs can regulate the secretion of gastrointestinal hormones such as glucagon-like peptide-1, which can inhibit postprandial appetite, slow down gastrointestinal motility, and increase insulin secretion (Tolhurst et al., 2012). Additionally, both GPR43 and GPR109a are expressed in the kidneys. Studies have shown that GPR43 deficiency impairs AMPK-α activity and insulin resistance in DKD patients (Lu et al., 2021). And dietary fiber can promote the production of SCFAs, which can reduce inflammation levels such as IL-6 and TNF-α, alleviate fibrosis-promoting factors such as collagen and TGF-β, and fight against glomerulosclerosis (Li et al., 2020). SCFAs are also HDACs inhibitors in the body, and they affect the expression of genes with multiple functions by inhibiting HDACs activity, which exhibits regulatory, anti-inflammatory, and antifibrotic activities (Wu et al., 2020). HDACs is present in human kidney tissue, and the progression of many kidney diseases is related to the acetylation modification of genes, playing a key role in diseases such as acute kidney injury (AKI), CKD, and DKD (Vinolo et al., 2011; Feng et al., 2018; Yang et al., 2020). Studies have shown that HDAC3 is abnormally active in fibrotic kidneys caused by unilateral ureteral obstruction (UUO) and aristolochic acid nephropathy (AAN), and its deletion can promote the expression of klotho to fight against kidney fibrosis (Chun, 2018; Chen et al., 2021). In DKD, HDAC5 is expressed in the kidneys of diabetic patients and animal models, and its activation exacerbates the epithelial-mesenchymal transition of renal tubular cells (Xu et al., 2021). Interestingly, studies have shown that the protective effect of short-chain fatty acids on the kidneys is exerted by activating GPCRs and inhibiting HDACs at the same time. It is currently unclear whether the protective effect of short-chain fatty acids on the kidneys is through GPCRs, HDACs, or both, and further research is needed to find out (Liu Y. et al., 2021).

Another important metabolite of the gut microbiota is bile acids. The liver produces primary bile acids, which include cholic acid, chenodeoxycholic acid, and others. After secretion into the intestine, the primary bile acids are converted to secondary bile acids, such as deoxycholic acid and lithocholic acid, through the action of the gut microbiota (Funabashi et al., 2020). Recently, it has been discovered that bile acid receptors, such as farnesoid X receptor (FXR) and G protein-coupled receptor 5 (TGR5) (Kuipers et al., 2004; Deutschmann et al., 2018), play a role in regulating obesity, diabetes, and nonalcoholic fatty liver disease by activating transcription factors related to lipid, cholesterol, and carbohydrate metabolism (Chávez-Talavera et al., 2017). FXR is the nuclear receptor for bile acids and is widely expressed in the intestine, liver, and kidney (Wang H. et al., 2018; Sun et al., 2021; Xu et al., 2022). It has an important regulatory role in various metabolic pathways and has been shown to improve obesity, liver damage, and bile stasis (Appelman et al., 2021; Panzitt and Wagner, 2021). Activation of FXR can improve systemic insulin resistance, lipid metabolism disorders, and organ changes in a type 2 diabetic kidney animal model (Han et al., 2021). In AKI, fatty acid oxidation (FAO) is inhibited, but overexpression of FXR can activate the FAO and peroxisome proliferator-activated receptor-γ (PPAR-γ) signaling pathways, improving FAO and reducing lipid accumulation in AKI (Xu et al., 2022). Patients with DKD and obesity-related glomerulopathy (ORG) had reduced expression of Takeda G-protein-coupled receptor 5 (TGR5) mRNA in kidney biopsy samples, which led to the metabolic disorders commonly seen in diabetes and obesity. Activation of TGR5 can regulate glucose and fatty acid metabolism, preventing DKD or ORG (Wang et al., 2016). In addition, low levels of TGR5 mRNA are associated with kidney disease progression. In renal tissue, TGR5 inhibits the Sphingosine-1-phosphate (S1P)/Sphingosine-1-phosphate receptor 2 (S1P2) signaling pathway, reduces the expression of Intercellular Adhesion Molecule-1 (ICAM-1), Transforming Growth Factor-beta 1 (TGF-β1), and Fibronectin (FN) in mesangial cells, and alleviates kidney fibrosis (Yang et al., 2016). It has been reported that the dual FXR/TGR5 agonist INT-767 can prevent mitochondrial dysfunction and oxidative stress, improve proteinuria, and prevent podocyte injury, mesangial expansion, and tubulointerstitial fibrosis in diabetic DBA/2J and db/db mice (Wang X. X. et al., 2018).

### 4.3 IgA nephropathy: immunity, infection and probiotic

In terms of the inflammation and immunity mechanisms of the gut-kidney axis, a co-occurrence analysis of keywords in the red and green clusters identified “immunity,” “immune response,” “infection,” “chronic inflammation,” and “IgA nephropathy” as important research hotspots that warrant further attention. Immunoglobulin A (IgA) is the most commonly produced antibody in the human body, secreted in mucosal tissue such as the palatine tonsils and the intestinal tract (Gutzeit et al., 2014; Liu et al., 2020; Isho et al., 2021). IgAN is characterized by the abnormal production of galactose-deficient immunoglobulin A1 (Gd-IgA1), which can lead to aberrant immune-inflammatory responses and cause damage to renal tissue (Berthoux et al., 2012). Deregulation of gut homeostasis, along with concomitant aberrant activation of gut immunity, can result in inflammatory bowel disease (IBD) and IgAN (Gevers et al., 2014; Kiryluk et al., 2014; Yang and Palm, 2020; Seikrit and Pabst, 2021). A previous study showed a significant association between IBD and IgAN, of which the gut microbiota is considered a key factor in pathogenesis and progression (Qing et al., 2022). Furthermore, the data strongly suggests that microbiota-dependent IgA is significantly increased in the serum of IgAN patients (Han et al., 2022). In particular, the microbiota in patients with IgAN differed from those in healthy controls, and dysbiosis of the gut microbiota has been suggested as an important factor in the overproduction of Gd-IgA1(Tang et al., 2022). Some studies suggest that bacterial infection may be a trigger for the onset of IgA nephropathy. In addition, bacterial infection may also cause an inflammatory response, increasing the risk of kidney damage in glomeruli. What’s more, infection may cause an immune system response, leading to abnormal production of Gd-IgA1, which deposits in the glomeruli of the kidneys (He et al., 2020). It is noted that although probiotics may be beneficial for IgA nephropathy, Probiotics are beneficial bacteria that can grow in the intestines and have a positive effect on the human body. Some animal experiments show that probiotics can improve gut dysbiosis and inflammatory reactions in IgA nephropathy (Tan et al., 2022). In conclusion, gut microbiota dysbiosis contributes to the superactivation of the immune response, which plays a crucial role in the development of IgAN.

### 4.4 Chronic kidney disease: uremic toxins

Uremic toxins is linked to the advancement of CKD, as the imbalance of gut microbiota accelerates the production of uremic toxins, including indoxyl sulfate (IS), p-Cresyl Sulfate (PCS), phenyl sulfate (PS) and trimethylamine N-oxide (TMAO). The reduced ability to excrete these toxins due to impaired renal clearance results in their accumulation in the gut of CKD patients, disrupting gut microbiota and leading to an increased production of uremic toxins in the gut (Skye and Hazen, 2016). The gut microbiota plays a well-known role in producing uremic toxins, with IS/PCS/PS/TMAO being formed by the gut microbial metabolic pathways. The production of IS occurs when a tryptophanase produced by gut microbiota catalyzes the conversion of tryptophan into indole. Indole is then converted into indoxyl phenol and IS in the host liver through the continuous action of cytochrome P450 2E1 and Sulfotransferase 1A1 (Devlin et al., 2016). On the other hand, gut bacteria produces a specific tyrosine phenol lyase that decomposes tyrosine into phenol and is further modified to produce PS or PCS (Kikuchi et al., 2019). Meanwhile, TMAO is formed when gut microbiota metabolizes choline, carnitine, and phosphatidylcholine to form trimethylamine, which is then oxidized to TMAO in the liver via flavin-dependent monooxygenase (FMO) (Aron-Wisnewsky and Clément, 2016). IS/PS/PCS are protein-bound toxins that form large molecular complexes with proteins in the blood, making it difficult to effectively clear them through conventional dialysis (Graboski and Redinbo, 2020). Although healthy individuals have low levels of uremic toxins, they can be more than 100 times higher in the circulation of kidney disease patients. In proximal tubule cells, IS can stimulate the expression of nuclear factor NF-κB and plasminogen activator inhibitor type 1, leading to renal toxicity and enhancing the expression of tissue inhibitor of metalloproteinase and TGF-β1, which are involved in renal tubulointerstitial fibrosis. Studies have shown that IS promotes the expression of inflammatory cytokine-related genes and activates pro-fibrotic genes in the kidney. The downregulation of Klotho expression, which promotes glomerulosclerosis and renal tubulointerstitial fibrosis, may be a contributing factor to this phenomenon. Additionally, IS enhances Klotho hypermethylation and promotes the progression of vascular calcification in CKD (Chen et al., 2016). Aryl hydrocarbon receptor (AHR) is a cytoplasmic ligand-activated transcription factor that can increase the production of cytokines associated with inflammation, thrombosis, and leukocyte adhesion. The activation of AHR by IS/PS/PCS can contribute to the development of CKD. The potential for AHR activation has been found to be higher in CKD patients and animal models and is associated with an increased risk of cardiovascular disease (Dou et al., 2018). The activation of AHR can cause progressive damage to glomerular and tubular cells, leading to glomerulosclerosis and tubulointerstitial fibrosis, thus exacerbating CKD (Liu J. R. et al., 2021). Furthermore, mice exposed to IS for 8 weeks will exhibit progressive renal dysfunction. IS can induce AHR nuclear translocation, increase Cytochrome P450 1A1 levels, and decrease the cell size and vitality of mouse foot cells (Dou et al., 2018). In the PS cohort of diabetic patients, the level of PS is significantly associated with both the baseline and predicted 2-year progression of microalbuminuria (Kikuchi et al., 2019). Similar to PS, IS, and PCS are associated with increased inflammatory biomarkers in patients with stage 3–4 CKD, such as glutathione peroxidase and IL-6. PCS is associated with severe renal tubular damage in 5/6 nephrectomized rats because it enhances oxidative stress and inflammatory cytokine levels, and elevated PCS levels are associated with worse prognosis in CKD patients (Liu et al., 2018), as demonstrated by a cohort study showing that free PCS has the highest correlation with cardiovascular outcomes in non-dialysis CKD patients. Free PCS may exert its cardiovascular toxicity by adversely affecting endothelial function (Glorieux et al., 2021). The association between the gut microbiota metabolite TMAO and cardiovascular disease has been a focus of related research (Aron-Wisnewsky and Clément, 2016), but TMAO also promotes the development of CKD and increases the risk of death from CKD (Tang et al., 2015). Participants with lowered TMAO showed a decrease in fasting blood glucose and a significant increase in insulin sensitivity, while participants with elevated TMAO showed a smaller improvement in fasting blood glucose and insulin sensitivity, providing strong evidence of the important role of TMAO in the development of diabetes (Heianza et al., 2019). A meta-analysis focusing on the relationship between TMAO and kidney disease indicated that the TMAO content in the peripheral circulation is significantly negatively correlated with the glomerular filtration rate of the subjects, and the TMAO content in late-stage kidney disease patients is significantly increased, indicating a close relationship between TMAO and kidney function damage (Zeng et al., 2021). Additionally, studies have found that supplementing mice with CKD models with iodomethylcholine, which selectively inhibits TMA lyase activity, can effectively suppress diet-induced increases in TMAO levels and subsequent renal tubulointerstitial fibrosis, thereby improving renal function damage and revealing the impact of TMAO on kidney function damage and related mechanisms (Gupta et al., 2020). In recent years, researchers have been focusing on the regulatory effect of gut microbiota on the clearance of uremic toxins. Lobel et al. reported that a high sulfur amino acid diet induces translationally modified microbial enzymes (S-sulfation), resulting in a reduction in the production of uremic toxins (Lobel et al., 2020). A meta-analysis suggests that supplementing with prebiotics and synbiotics can significantly lower circulating PCS concentrations in CKD and slow the progression of kidney disease (Rossi et al., 2016).

## 5 Limitations

In contrast to traditional reviews, our study that employs bibliometric tools provides a more detailed and quantitative picture of research focus, trends, and collaborations, which yields better insights into the evolving future of the gut-kidney axis. However, we must recognize certain limitations. Firstly, we only included publications in the WOSCC database, which excludes non-English publications, potentially creating publication bias. Secondly, citation data is subject to time constraints, with earlier papers being cited more frequently than recent ones. This may result in some influential papers not receiving due attention because of their short duration. Thirdly, we did not screen or organize the literature retrieved by the database search, which may lead to the inclusion of literature that is not closely related to this field, resulting in false-positive results. Furthermore, some review articles, rather than original research, may have caused bias due to frequent citation. What’s more, in the discussion section, we primarily focused on DKD, IgAN, and CKD as the core areas of research and highlighted the current research hotspots. However, it is essential to recognize that the gut-kidney axis holds potential research significance in the context of other kidney diseases. Despite these limitations, we believe our findings provide a more comprehensive examination of the gut-kidney axis research, which could offer valuable insights into current and future research in this field.

## 6 Conclusion

This is the first comprehensive study on the research focus and future trends of the gut-kidney axis, particularly in the context of the gut microbiota and kidney disease research. Overall, we found that the number of papers in this field has been rapidly increasing since the gut-kidney axis was first introduced in 2011. The United States is still the pioneer and most influential in this field, with most of the highest publishing institutions located in the United States. China is catching up and needs to increase the impact of its papers and strengthen global cooperation. Journal analysis found that papers on the gut-kidney axis have gradually shifted from toxin-related journals to metabolism-related journals, reflecting a development trend in the field. Clustering analysis of keywords found that the important research focuses are “immunity,” “inflammation,” “metabolism,” and “uremic toxins,” which reflect the research foundation of this field. Future research should focus on topics such as “hyperuricemia,” “gut microbiota,” “diabetes,” “trimethylamine n-oxide,” “iga nephropathy,” “acute kidney injury,” “chronic kidney disease,” “inflammation,” which have been identified as the forefront of this field. These timely analytical results provide a new perspective on the treatment of kidney disease, help researchers choose appropriate publishing journals, find potential collaborators, understand hotspots and frontiers, and promote the development of this field.
