# Supplementary figures and images for: Bibliometric visualization analysis of gut-kidney axis from 2003 to 2022
Source: Front Physiol. 2023 Jun 9;14:1176894. doi: 10.3389/fphys.2023.1176894 (PMC10287975; doi:10.3389/fphys.2023.1176894)

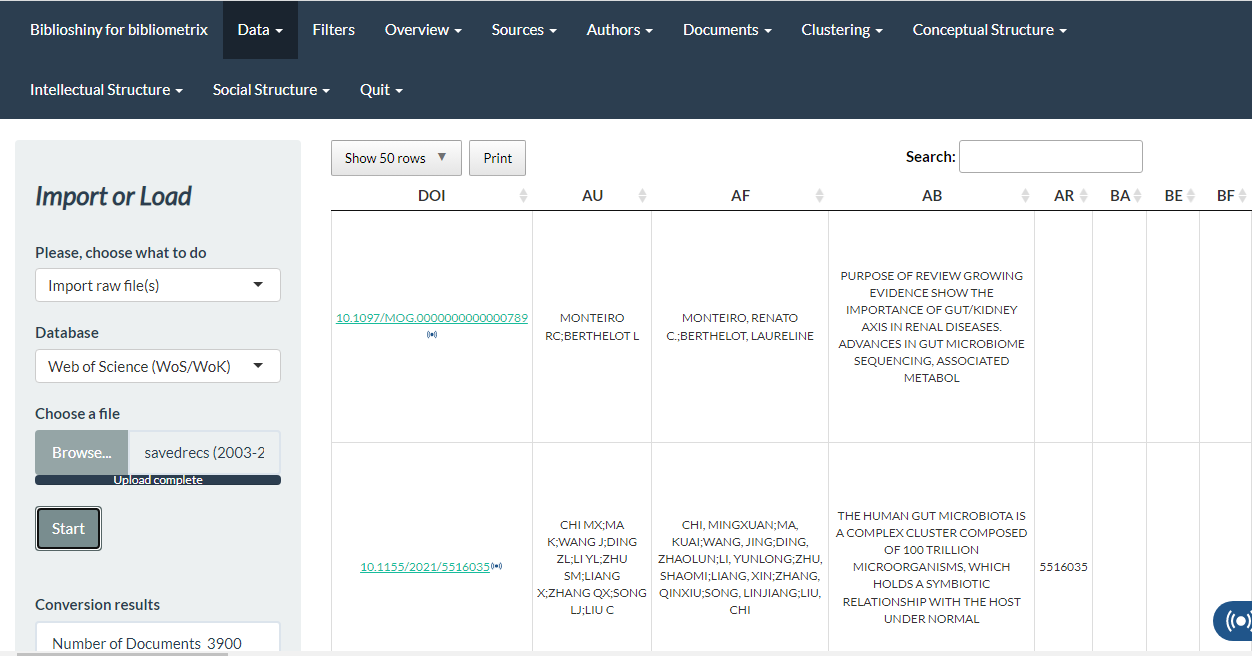

Supplement: Supplementary file 1 [file DataSheet1.zip › supplementary material/Import to Biblioshiny for bibliometrix.png]

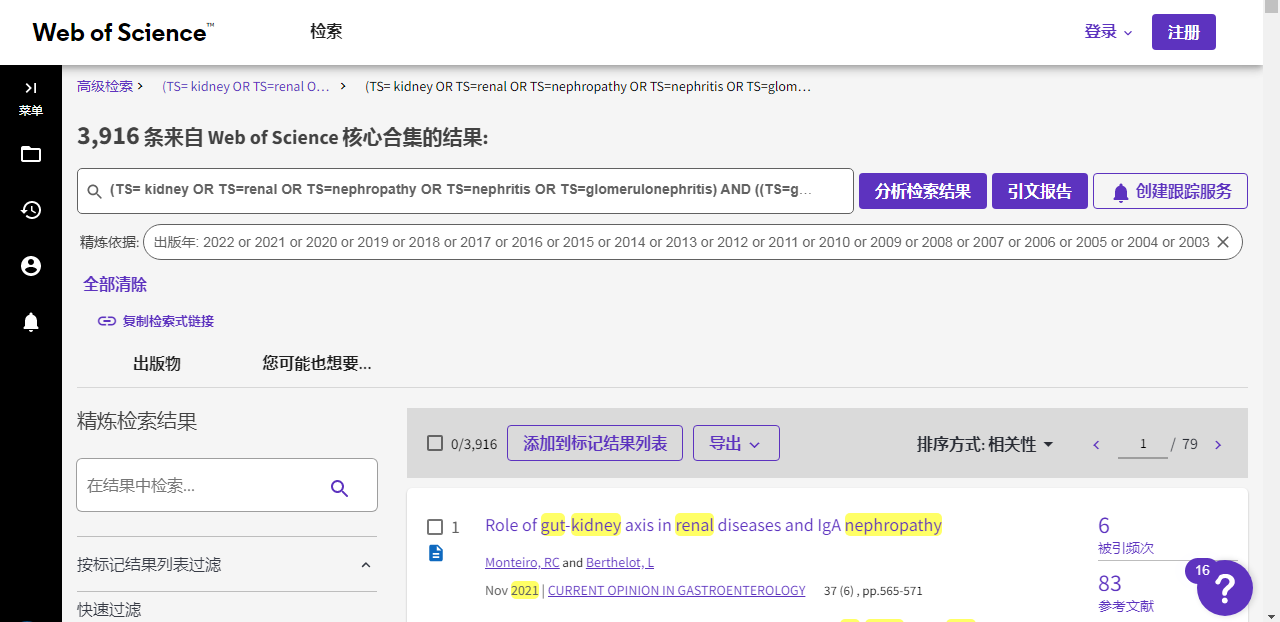

Supplement: Supplementary file 1 [file DataSheet1.zip › supplementary material/WOSCC database searching result.png]
